# Bilateral Adrenal Myelolipoma and Breast Cancer in a Patient With Congenital Adrenal Hyperplasia

**DOI:** 10.7759/cureus.54784

**Published:** 2024-02-23

**Authors:** Zahraa M. M. Zeer, Mahmoud Noman, Almotazbellah M Alzeer, Yamama Mahamid, Malak Abu Moch, Alaa Atwaneh

**Affiliations:** 1 Faculty of Medicine, Al-Quds University, Jerusalem, PSE; 2 Endocrinology, Augusta Victoria Hospital, Jerusalem, PSE

**Keywords:** adenoma, incidental mass, breast cancer, myelolipoma, adrenal glands

## Abstract

Adrenal myelolipoma is a rare, benign tumor of the adrenal gland, typically non-functional, asymptomatic and unilateral. With the increased use of radiological imaging, it has been discovered more frequently as incidental mass. It is common to occur concurrently with hormonal dysfunction conditions like congenital adrenal hyperplasia. However, there are few previous reported cases of malignancy concomitant with adrenal myelolipoma. We present a case of a 33-year-old patient diagnosed with congenital adrenal hyperplasia since birth. She was diagnosed with giant bilateral adrenal myelolipoma incidentally during the investigation done for staging her breast cancer. To the best of our knowledge, this is the second reported case of breast cancer concomitant with adrenal myelolipoma. Although this entity is very rare, physicians should be familiar with such rare adrenal masses and their associations in order to manage them appropriately.

## Introduction

Adrenal myelolipoma is a benign tumor composed mainly of hematopoietic cells interspersed between mature adipose tissue. They are most commonly nonfunctioning, unilateral and asymptomatic [1]. Although they are very rare, their detection incidence has become more common with the widespread use of abdominal imaging for other reasons [2]. Hormonal evaluation is indicated in order to exclude any hormonal disorders as congenital adrenal hyperplasia and Cushing’s syndrome which are commonly associated with adrenal myelolipoma [3]. In addition, in the literature, few cases of adrenal myelolipoma are reported concomitant with cases of malignancies [4]. In our presented case, a 33-year-old patient diagnosed with congenital adrenal hyperplasia since birth was diagnosed to have giant bilateral adrenal myelolipoma incidentally during the investigation done for staging her breast cancer.

## Case presentation

In October 2018, our oncology facility admitted a 33-year-old female patient subsequent to her breast cancer diagnosis. Her medical history includes congenital adrenal hyperplasia, specifically virilizing congenital adrenal hyperplasia (21-hydroxylase deficiency), diagnosed at birth. This condition, characterized by ambiguous genitalia and clitoromegaly, has been managed through a therapeutic regimen involving glucocorticoids, mineralocorticoids, and reconstructive surgical interventions. At the age of 12, she underwent vaginoplasty, which was complicated by a urogenital fistula identified during cystoscopy. Subsequently, at the age of 26, a second vaginoplasty, in conjunction with vaginal dilatation, was performed. Significantly, the patient's siblings, both a brother and a sister, also share this congenital adrenal hyperplasia condition.

Histopathological examination from a tru-cut needle biopsy confirmed the presence of left breast invasive ductal carcinoma. A whole-body CT scan detected suprarenal cystic lesions measuring 6.9x6.7x2.7 on the right side and 8.9x8.9x3 on the left side without signs of metastatic spread. The patient underwent lumpectomy with axillary clearance found no metastatic carcinoma in the harvested reactive lymph nodes, revealing Stage IA (T1.N0.M0) breast cancer, with positive estrogen, progesterone, and HER2neu receptors. Following this, she received four cycles of adjuvant doxorubicin cyclophosphamide (AC) chemotherapy and underwent adjuvant radiotherapy (16 fractions). Then she was maintained on adjuvant hormonal therapy with tamoxifen at a daily dose of 20 mg orally and Herceptin (trastuzumab) at 600 mg every 21 days.

**Figure 1:**
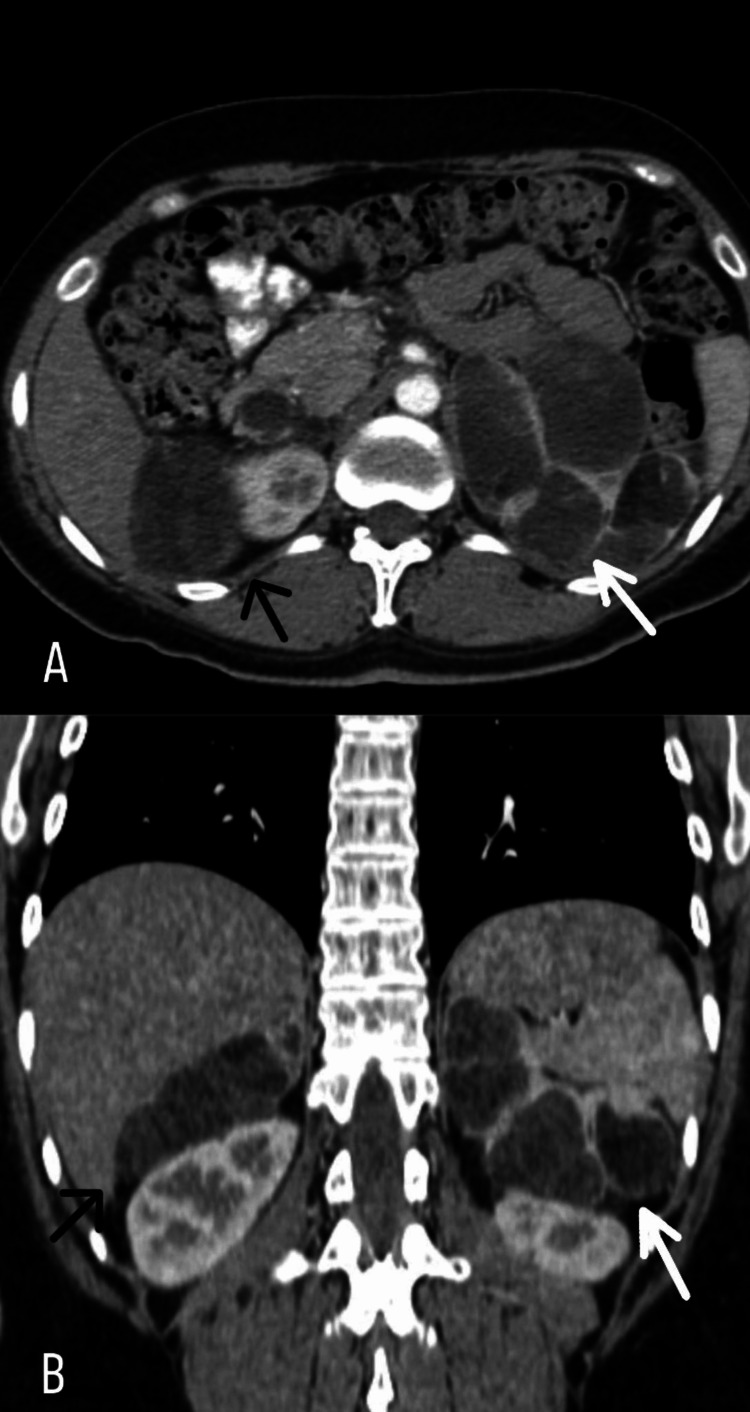
A: axial view, B: coronal view. Computed tomography (CT) scan uncovered bilateral adrenal masses characterized by a heterogeneous and multicystic nature. The right mass measured 8x5cm (black arrow) and the left mass measured 10x7.5cm (white arrow).

In June 2020, a subsequent CT scan revealed no signs of disease but identified hepatic cystic lesions. In December 2020, two years post breast cancer diagnosis, a follow-up CT scan uncovered bilateral adrenal masses characterized by a heterogeneous and multicystic nature. The left mass measured 10x7.5cm, and the right mass measured 8x5cm (Figure 1), with the primary diagnostic considerations encompassing bilateral pheochromocytoma and potential metastatic deposits.

A hormonal panel exhibited an elevated recumbent aldosterone level at 537 pg/ml (reference: 10-160), while plasma metanephrine (0.36 nmol/L, reference <0.50) and normetanephrine (0.82, reference <0.90) were within normal ranges. Additionally, dehydroepiandrosterone was 166 (reference: 98-340), potassium was 4.57mmol/l (reference: 3.50-5.30), adrenocorticotropic hormone (ACTH) was 5.28pg/ml (reference: 7.20-63.30), and the morning cortisol level was low at 2.33 ug/dl (reference: 4.82-19.50).

**Figure 2:**
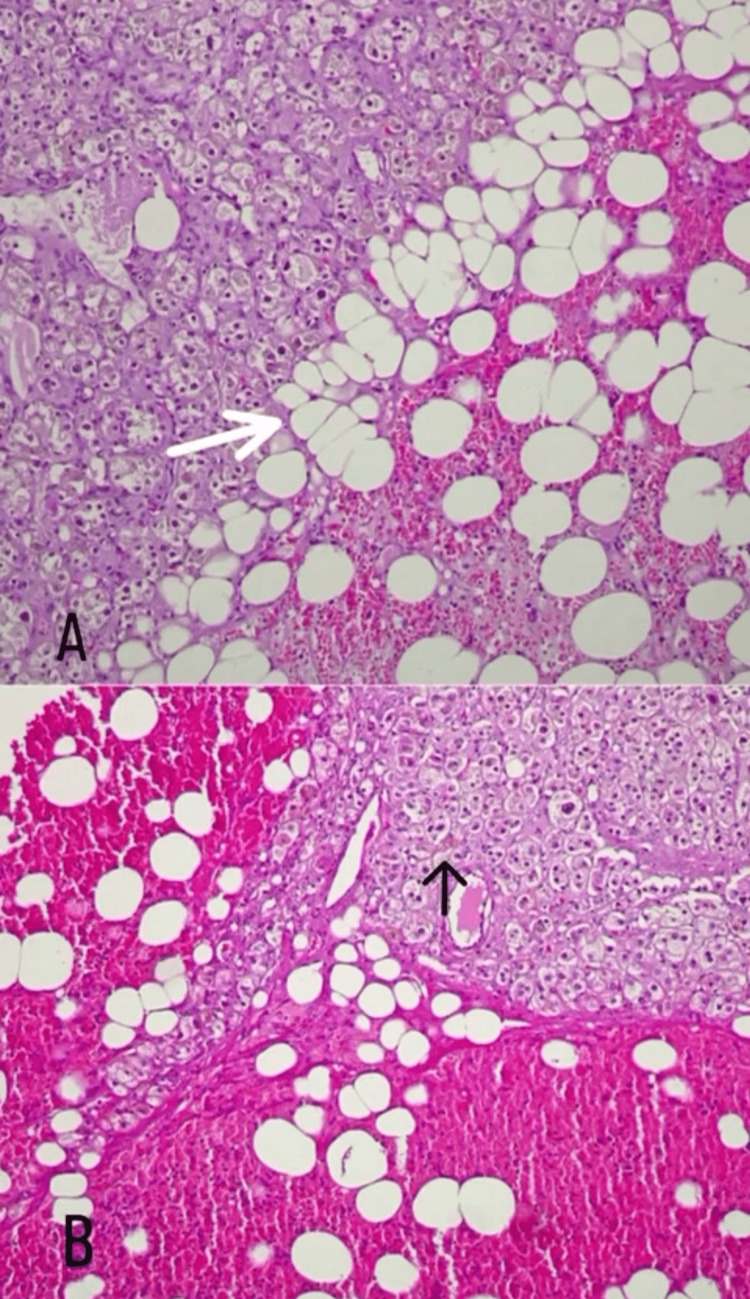
Microscopic features. A: (10× magnification) formalin-fixed paraffin-embedded sections showing sheets of mature adipocytes within adrenal gland tissue (white arrow). B: (10 × magnification) formalin-fixed paraffin-embedded sections from adrenal gland tissue and hematopoietic cells (black arrow) with mature adipocytes.

Given these findings, the patient underwent bilateral adrenalectomy in April 2021. The removal of both adrenal masses proceeded without complications. The patient received stress hydrocortisone both pre- and post-surgery and continued with a regular dose of hydrocortisone at 50mg four times a day. The gross pathological examination illustrated bilateral adrenal gland masses consisting of bright-yellow adipose-like tissue with hemorrhagic foci, measuring 11x9.5 cm (left adrenal gland mass) and 9x5 cm (right adrenal gland mass). Microscopic examination revealed bilateral myelolipoma with no evidence of malignancy (Figure 2).

Subsequent to the surgery, the patient underwent a second hormonal workup, which returned within normal parameters. She is currently under regular follow-up, showing no evidence of disease.

## Discussion

Adrenal myelolipoma was first described in 1905 by Gierke as a lesion constituted of mature fat tissue mixed with erythroid and myeloid tissues [5]. It constitutes about 3.3-6.5% of all the adrenal masses with the second most common adrenal incidentalomas after adrenal adenoma [6] and accounts for 6-16% from all the adrenal incidentaloma detected radiologically. They are most common to be unilateral (95% cases) [2] with right-sided predilection three times more than the left side [7]. Extra adrenal myelolipoma has also been reported in multiple sites like mediastinum, thorax, retroperitoneum and pelvic cavity [4]. They are most commonly smaller than 4cm in size. We call them giant when they exceed 8cm [8]. The largest reported unilateral adrenal myelolipoma measuring 31x24.5x11.5cm and weighed 6 kg. In the case of bilateral masses, left-sided masses are usually larger than the right-sided masses and this asymmetric growth was speculated by constrain space due to the liver in the right side. The largest bilateral adrenal myelolipoma reported was 23x11x19cm and weighed 5.8 kg in the left and 15x13x6.8 and weighed 0.78 on the right [5]. Adrenal myelolipomas are stable in size or have slow growth. In a retrospective study of 321 patients with adrenal myelolipoma, it was found that the annual growth rate ranges from 6mm to 14mm [9]. Adrenal myelolipomas are commonly found between the fifth and seventh decade of life with a median age of 62 years with an equal gender distribution [10]. Unlike the previous reported cases, our patient is a 33-year-old female who presented with bilateral adrenal myelolipoma.

The exact etiopathogenesis is not definitely known but there are many theories that could explain it. The most common one is metaplasia of the reticuloendothelial cells as a result of stimuli such as necrosis and inflammation [2]. Another hypothesis claims that adrenal cortex secretes mediators as a result of inflammation from adipocytes growth in the endothelium that leads to recruitment of hematopoietic progenitors. Other theories are related to extramedullary hematopoiesis from bone marrow cells transferred during embryogenesis [7].

Adrenal myelolipoma is a nonfunctional mass but it can be associated with endocrinological disorders such as Cushing’s disease, cortical adenoma, ganglioneuroma, carcinoma, pheochromocytoma, Conn’s syndrome, diabetes mellitus, overproduction of dehydroepiandrosterone and congenital adrenal hyperplasia (CAH) due to 21 hydroxylase deficiency or 17 𝛼-hydroxylase deficiency. CAH and Cushing’s disease are the two most common associations [11]. It was reported that 10% of 440 patients with congenital adrenal hyperplasia had myelolipoma in a previous literature review [12].

Elevated level of ACTH was hypothesized as one of the theories for the formation of adrenal myelolipoma. This elevated level is believed to stimulate the metaplasia of adrenocortical cells to myelolipoma. These cases of adrenal myelolipoma were most commonly reported in patients with congenital adrenal hyperplasia especially those who are not treated and exposed to long periods of elevated ACTH which supports this theory [13]. Myelolipoma in these patients is noted to have melanocortin 2 receptor (MC2R) and androgen receptors and these factors are responsible for the continuous growth of these lesions [14]. It was also reported to have adrenal myelolipoma in patients with small cell lung carcinoma who have ectopic ACTH which also support the theory of ACTH inducing myelolipoma formation. On the other hand, there are other studies that does not align with this theory. Myelolipoma cases were reported in patients with ACTH-independent Cushing’s syndrome patients. In addition, one study showed that ACTH receptors are not overexpressed in myelolipoma as revealed by the histological analysis [5]. 

There are few cases reported in the literature with adrenal myelolipoma concomitant with malignancies such as adrenal carcinoma which was reported as the most common associated malignancy, bladder carcinoma and collecting duct carcinoma of the kidney. To the best of our knowledge, this is the second reported case of simultaneous breast cancer with adrenal myelolipoma. On the other hand, breast cancer is considered the most common cause of adrenal metastasis after lung cancer with higher prevalence of infiltrative ductal carcinoma than invasive ductal carcinoma. This emphasizes the importance of diagnosis adrenal masses in oncologic patients and evaluate the possibility of metastasis. Radiographic diagnosis plays an important role in these cases as there are many radiological features that correlate with malignant adrenal lesions. These features include: a mass size >4cm, a cutoff value <10 HU, irregular boundaries, heterogeneous enhancement, and <40% of the relative percentage washout [4].

The most common presentation for adrenal myelolipoma is asymptomatic, incidental finding identified during investigation for other reasons, but it can cause flank pain, hypochondrial pain [6], fever, constipation, hematuria and weight loss [15]. Compression effect may be seen on the peritumor tissue which causes renovascular hypertension [7]. Large lesions may be complicated by necrosis, rupture and hemorrhage. The risk of hemorrhage increases when adrenal myelolipoma exceeds 4cm in size and if fat component constitutes more than 50% of the mass [6].

In order to exclude any hormonal disorders which could be congenital, secondary endocrinological disorder or secretory neoplasm, hormonal evaluation should be done as a part of the workup for incidental adrenal myelolipoma [7]. This includes renin, aldosterone, metanephrine level, 1 mg overnight dexamethasone suppression test, ACTH, free cortisol, vanillylmandelic acid, homovanillic acid and 17-alpha hydroxyprogesterone level to exclude congenital adrenal hyperplasia [3]. 

US, CT and MRI can help with the diagnosis but CT is reported to be the most sensitive diagnostic method for adrenal myelolipoma. Hyperechoic and hypoechoic regions can be present in the same lesion. The fat component appears as hyperechoic regions and the myeloid component appears as hypoechoic regions [7]. Calcification was reported in 20% of the cases of adrenal myelolipoma in CT [16]. MRI is also used for distinguishing myelolipoma from other adrenal masses through making accurate quantitative evaluation of adrenal masses by doing comparisons between unenhanced and contrast-enhanced scans. When doing unenhanced MRI scans on T1- and T2-weighted images, masses with high fat proportion will show high signal intensity. After contrast enhancement, nothing will be changed on the signal intensity for the adipose-rich masses [17].

The fat component appears as a low density with less than -30 Hounsfield units. Although the presence of the macroscopic fat is the main diagnostic feature, it is not exclusive for adrenal myelolipoma as adenoma with myelolipomatous degeneration and adrenal cortical carcinoma may also show macroscopic fat. However, some authors suggest that if the fat component constitutes more than 50% of the lesion, it is considered to be myelolipoma. Some features should raise the possibility of rare fat-containing malignancies such as irregular margins, heterogenicity, and local invasion [18]. Histologically, these masses constitute variable amounts of mature adipose tissue [3] mixed with island of hematopoietic elements including erythroid, myeloid, lymphatic cells as well as megakaryocytes [5]. The differential diagnoses of adrenal myelolipoma include fat-containing adrenal masses which include lipomas, lipomyosarcomas, teratomas, angiomyolipoma and fatty adrenal adenoma [11]. 

As there are no specific guidelines for the management of bilateral adrenal myelolipoma, it should be done case-by-case based on the presence of symptoms, the size of the lesion, any suspicion for malignancy and the rate of growth during follow-up period [7].

If the lesion is asymptomatic, less than 7cm in size, it should be managed by monitoring the growth over one to two years. On the other hand, surgical management is indicated if there is any suspicion for malignancy, significant growth in the follow-up period, the presence of hormonal activity or if the size of the mass is greater than 7cm. This could be done by laparotomy or laparoscopy [3]. Laparoscopic approach is superior to the open method as it has lower morbidity which includes less surgical site infection with faster recovery and hospital discharge. However, laparotomy is advised in cases of large lesions more than 10cm, in cases of adhesion or infiltration to surrounding structures [15]. The best strategy for bilateral adrenalectomy is the two-stage surgery in order to avoid adrenal crisis and minimize the postoperative complications [17]. Patients with bilateral adrenalectomy need to be on steroid replacement therapy [12].

In the case of bilateral adrenal myelolipoma, there are no reported cases for emergent intervention for bleeding due to spontaneous rupture or traumatic events. On the other hand, for unilateral adrenal myelolipoma, it was reported that embolization was used for spontaneous bleeding followed by elective adrenalectomy. In addition, adrenalectomy for the symptomatic lesion was performed in cases of rupture. It is worth mentioning that these complications were all encountered with lesions bigger than 7cm [3]. In general, adrenal myelolipoma does not recur and as reported in the literature it has a recurrence-free survival of 15 years [1]. 

## Conclusions

In conclusion, this case emphasizes the importance of considering adrenal myelolipoma as a potential diagnosis of incidental adrenal masses in patients with a history of CAH. Early diagnosis and prompt surgical intervention through comprehensive diagnostic approaches in conjunction with multidisciplinary team efforts are required in order to prevent complications associated with giant adrenal myelolipoma. Further research studies are needed for better understanding of the pathogenesis of adrenal myelolipoma, its associated conditions including hormonal disorders or malignancies and the required optimal management. 
